# The neuronal guidance protein netrin-1 reduces alveolar inflammation in a porcine model of acute lung injury

**DOI:** 10.1186/cc9301

**Published:** 2010-10-22

**Authors:** Christian Mutz, Valbona Mirakaj, Dierk A Vagts, Phillipp Westermann, Kristina Waibler, Klemens König, Thomas Iber, Gabriele Nöldge-Schomburg, Peter Rosenberger

**Affiliations:** 1 Department of Anesthesiology and Intensive Care Medicine, University Hospital Rostock, Rostock University, Schillingallee 35, Rostock 18057, Germany; 2 Department of Anesthesiology and Intensive Care Medicine, University Hospital Tübingen, Eberhard-Karls University, Hoppe Seyler Strasse 3, Tübingen 72076, Germany; 3 Clinic of Anesthesiology, Intensive Care Medicine and Pain Therapy, University Hospital Frankfurt am Main, Goethe University, Theodor Stern Kai 7, Frankfurt am Main 60590, Germany

## Abstract

**Introduction:**

Acute lung injury (ALI) is an inflammatory disorder of pulmonary or extrapulmonary origin. We have previously demonstrated that netrin-1 dampens murine ALI, and in an attempt to advance this finding into future clinical practice we evaluated whether netrin-1 would reduce alveolar inflammation during porcine ALI.

**Methods:**

This was a controlled *in vivo *experimental study in pigs. We induced ALI through lipoploysaccharide (LPS) infusion (50 μg/kg) for 2 hours. Following this, we exposed animals to either vehicle, intravenous netrin-1 (netrin-1 i.v.) or inhaled netrin-1 (netrin-1 inh.). Serum samples and bronchoalveolar lavage (BAL) were obtained to determine levels of tumor necrosis factor-α (TNF-α), interleukin (IL)-1β, interleukin-6 and interleukin-8 at baseline and 6 hours following treatment. Myeloperoxidase activity (MPO) and protein levels were determined in the BAL, and tissue samples were obtained for histological evaluation. Finally, animals were scanned with spiral CT.

**Results:**

Following LPS infusion, animals developed acute pulmonary injury. Serum levels of TNF-α and IL-6 were significantly reduced in the netrin-1 i.v. group. BAL demonstrated significantly reduced cytokine levels 6 hours post-netrin-1 treatment (TNF-α: vehicle 633 ± 172 pg/ml, netrin-1 i.v. 84 ± 5 pg/ml, netrin-1 inh. 168 ± 74 pg/ml; both *P *< 0.05). MPO activity and protein content were significantly reduced in BAL samples from netrin-1-treated animals. Histological sections confirmed reduced inflammatory changes in the netrin-1-treated animals. Computed tomography corroborated reduced pulmonary damage in both netrin-1-treated groups.

**Conclusions:**

We conclude that treatment with the endogenous anti-inflammatory protein netrin-1 reduces pulmonary inflammation during the initial stages of ALI and should be pursued as a future therapeutic option.

## Introduction

Acute lung injury (ALI) is a devastating disorder of the lung with an incidence of approximately 17.5/100,000 per year and is associated with high mortality [1,2]. The hallmarks of ALI include the disruption of the alveolar-capillary barrier, the extravasation of fluid from the vascular space causing pulmonary edema and the infiltration of leukocytes into the alveolar space [3,4]. Oxygenation is impaired, and the acute respiratory distress syndrome (ARDS) develops when the oxygenation index deteriorates to a PaO_2_/FiO_2 _quotient below 200 [2]. The importance of these inflammatory changes for the pathophysiology of ALI is supported by the finding that experimental lung injury can be substantially reduced through the depletion of neutrophils [5]. During the initial stages of ALI, neutrophils migrate into the alveolar space in response to secreted cytokines. Furthermore, cytokines increase the activation of alveolar macrophages and aggravate the intra-alveolar inflammation that might become self-propagating within the pulmonary tissue [5,6]. Several investigators have described the importance of this process by identifying a correlation between alveolar cytokine levels and the severity of pulmonary organ injury [7-11]. As a result, pulmonary oxygen exchange is compromised [12,13]. Strategies for the treatment of ALI are aimed at improving pulmonary function through reducing the extent of pulmonary inflammation [14,15].

Netrin-1 was initially described in the context of central nervous system (CNS) development, where it influences axonal migration and guides CNS development [16-18]. But netrin-1 is also expressed outside the CNS in the intestine, kidney and lung, where it controls the trafficking of leukocytes from the vascular space [19,20]. During periods of intestinal hypoxia, netrin-1 is induced through hypoxia inducible factor 1-α and dampens local inflammation of the large intestinal surface [21]. This is in large part dependent on the coexpression of the adenosine 2B receptor (A2BAR), a receptor that is transcriptionally induced during hypoxia and inflammation [22,23]. Similarly to the intestinal organs, the pulmonary surface is composed of a large mucosal lining. This mucosal surface of the lung is characterized by a close proximity of the vascular and the alveolar space building the alveolar-capillary barrier. We were recently able to demonstrate that netrin-1 is expressed in endothelial and epithelial cells within pulmonary tissue and is significantly repressed during inflammatory and mechanical lung injury. Furthermore, employing animals with endogenous repression of netrin-1, we demonstrated increased pulmonary damage in these animals compared to littermate controls and that a substitution with exogenous netrin-1 resulted in a significant reduction of lung injury [20]. The protective role of netrin-1 in our murine model was also dependent on the A2BAR corroborating the protective potential of the A2BAR during lung injury [23].

Given the anti-inflammatory potential of neuronal guidance protein netrin-1 and the important role of a control of pulmonary inflammation during ALI, we investigated whether our previous findings could be translated further and attempted to bring netrin-1 one step forward to a potential clinical use. For this purpose, we decided to use systemic and local netrin-1 administration to find the potentially best application for future therapeutic evaluation. We hypothesized that netrin-1 might be able to influence the extent of pulmonary inflammation and leukocyte activity within the alveolar space in a large animal model of ALI. This study was therefore aimed at improving our understanding of netrin-1 for a possible translation from mice to humans.

## Materials and methods

### Research animals

The study was approved by the local Ethics Committee on Animal Research (Landesamt für Landwirtschaft Rostock, Approval LALLF M-V/TSD/7221.3-1.1-038/08) and in concordance with the Helsinki convention. Female domestic pigs with an age of 3-4 months were used for the experimental procedure. The average weight of animals used in each group is provided in Table 1.

**Table 1:** Systemic hemodynamic measurements and pulmonary hemodynamic measurements

Parameter	Group		Time	
		
		0 h	4 h	6 h
Weight (kg)	Control	30.4 ± 0.69		
	LPS + Vehicle	31.0 ± 0.58		
	LPS + Netrin i.v.	30.2 ± 0.20		
	LPS + Netrin inh.	29.8 ± 0.49		

CI (ml min^-1 ^kg^-1^)	Control	166 ± 3.0	167 ± 5.1	156 ± 10.4
	LPS + Vehicle	170 ± 5.1	136 ± 10.5	122 ± 7.3*
	LPS + Netrin i.v.	163 ± 9.2	128 ± 11.9	114 ± 9.4*
	LPS + Netrin inh	160 ± 3.4	130 ± 11.9	118 ± 8.4*

MAP (mmHg)	Control	68 ± 3.7	77 ± 4.9	84 ± 3.7
	LPS + Vehicle	75 ± 6.0	89 ± 3.2	119 ± 6.4*
	LPS + Netrin i.v.	78 ± 6.5	97 ± 6.9*	124 ± 7.0*
	LPS + Netrin inh.	86 ± 6.7	93 ± 2.7	118 ± 4.5*

MPAP (mmHg)	Control	16 ± 1.4	17 ± 1.4	17 ± 1.8
	LPS + Vehicle	17 ± 0.7	31 ± 1.8*	31 ± 2.0*
	LPS + Netrin i.v.	18 ± 0.8	31 ± 2.0*	34 ± 2.3*
	LPS + Netrin inh	16 ± 0.5	28 ± 2.5*	31 ± 3.3*

SVR (dyne*s*cm5 kg^-1^)	Control	34 ± 1.6	39 ± 3.7	47 ± 5.6
	LPS + Vehicle	36 ± 3.6	54 ± 3.8	80 ± 8.1*
	LPS + Netrin i.v.	41 ± 4.6	66 ± 7.8*	95 ± 11.4*
	LPS + Netrin inh	47 ± 4.2	64 ± 6.2*	88 ± 7.0*

PVR (dyne*s*cm5 kg^-1^)	Control	6.5 ± 0.6	6.7 ± 0.4	7.2 ± 0.7
	LPS + Vehicle	6.6 ± 0.4	16.6 ± 2.3*	16.9 ± 1.4*
	LPS + Netrin i.v.	7.1 ± 0.5	16.6 ± 2.4*	20.6 ± 3.0*
	LPS + Netrin inh.	6.8 ± 0.4	15.5 ± 2.8*	18.9 ± 2.1*

PCWP (mmHg)	Control	3.9 ± 0.4	3.9 ± 0.5	4.0 ± 0.5
	LPS + Vehicle	3.2 ± 0.6	5.1 ± 0.9	6.6 ± 1.9*
	LPS + Netrin i.v.	4.4 ± 0.3	7.0 ± 0.5*	8.0 ± 0.5*
	LPS + Netrin inh	4.4 ± 0.5	6.8 ± 0.5*	7.6 ± 0.5*

ITBVI (ml kg^-1^)	Control	33.3 ± 1.5	33.0 ± 0.7	30.8 ± 1.6
	LPS + Vehicle	31.9 ± 1.8	28.0 ± 1.3	32.0 ± 3.0
	LPS + Netrin i.v.	34.4 ± 3.1	31.9 ± 2.0	35.5 ± 2.1
	LPS + Netrin inh.	32.1 ± 1.3	29.0 ± 0.8	33.0 ± 0.6

EVLWI (ml kg^-1^)	Control	5.5 ± 0.4	6.0 ± 0.6	6.5 ± 0.5
	LPS + Vehicle	5.7 ± 0.7	9.1 ± 2.1	9.7 ± 1.3*
	LPS + Netrin i.v.	5.1 ± 0.7	7.5 ± 0.4	11.9 ± 1.4*
	LPS + Netrin inh.	4.8 ± 0.4	6.3 ± 0.7	8.1 ± 1.3*

Measurements are compared in the control, vehicle and netrin-1 treatment groups 4 hours and 6 hours after the end of treatment (data are means ± SEM and indexed in body weight; *P *< 0.05; *versus control group). Cardiac index (CI), mean arterial pressure (MAP), mean pulmonary arterial pressure (MPAP), systemic vascular resistance index (SVR), pulmonary vascular resistance index (PVR), pulmonary capillary wedge pressure (PCWP), central venous pressure (CVP), intrathoracic blood volume index (ITBVI), extravascular lung water index (EVLWI).

### Anesthesia

Animals were premedicated with 8 μg kg^-1 ^intramuscular (i.m.) azaperone (Janssen-Cilag, Neuss, Germany), 6.0 mg kg^-1 ^ketamine (Bela-Pharm, Vechta, Germany) and 0.25 mg kg^-1 ^midazolam (Merckle, Blaubeuren, Germany) after overnight fasting and receiving water ad libitum. After an intravenous line was placed, anesthesia was induced intravenously (i.v.) with 3 μg kg^-1 ^fentanyl (Janssen-Cilag, Neuss, Germany), 1.6 mg kg^-1 ^ketamine (DeltaSelect, Pfullingen, Germany), 0.3 μg kg^-1 ^flunitrazepam (Roche Pharma, Grenzach, Germany) and 0.3 mg kg^-1 ^pancuronium (Hikma Farmaceutica, Reute, Germany). The trachea was intubated and anesthesia maintained by continuous i.v. infusion of flunitrazepam 85 μg kg^-1 ^h^-1^, 0.2 mg kg^-1 ^h^-1 ^pancuronium and 7 mg kg^-1 ^h^-1 ^ketamine. Mechanical ventilation was provided by pressure-controlled ventilation with a Servo 900 ventilator (Siemens, Munich, Germany). The ventilation protocol used a tidal volume of 10 ml/kg body weight and a positive end expiratory pressure of 3 cm H_2_O.

### Instrumentation

After placement of a 3-lm central venous catheter (Certofix; Braun, Meslungen, Germany) and an 8.5-F introducer (Arrow, Erding, Germany) into the right internal jugular vein, a Swan-Ganz thermodilution catheter (Edwards Laboratories, Irvine, CA, USA) was introduced into the pulmonary artery. Next, the right femoral artery was cannulated with a 4.5-Fr introducer set (Arrow, Erding, Germany), and a COLD catheter (Pulsion Medical Systems, Munich, Germany) was advanced 30 cm to reach the distal aorta. A suprapubic cystofix catheter (Braun, Melsungen, Germany) was inserted for measurement of diuresis. To provide adequate fluid replacement, animals received full-electrolyte solution 10 ml kg^-1 ^h^-1 ^i.v. (Jonosteril; Fresenius, Bad Homburg., Germany) during the study period.

### Measurement and calculations

All intravascular catheters were connected to pressure transducers, and signals were recorded online (PONEMAH, St. Paul, MN, USA). Measured variables included heart rate (HR), mean arterial pressure (MAP), central venous pressure (CVP), pulmonary artery pressure (PAP) and pulmonary capillary wedge pressure (PCWP). Equations for derived variables (e.g., systemic vascular resistance (SVR), pulmonary vascular resistance (PVR), systemic oxygen delivery (DO_2l_)) were as described previously [24,25]. Cardiac output was determined by thermodilution technique (Baxter, Unterschleißheim, Germany). The mean value of three injections of 10 ml of ice-cooled saline was considered to estimate actual cardiac output. Intrathoracic blood volume (ITBV) and extravascular lung water (EVWL) were determined with a double-indicator dilution technique using the COLD catheter.

### Bronchoscopy

At the described time points, bronchoscopy was performed by identifying the left lobe of the lung with a standard bronchoscope. Bronchoalveolar lavage was performed using 10 ml of normal saline. The solution was then centrifuged, and the supernatant was obtained for further analysis.

### Endotoxin Infusion and Experimental Protocol

**Figure 1:**
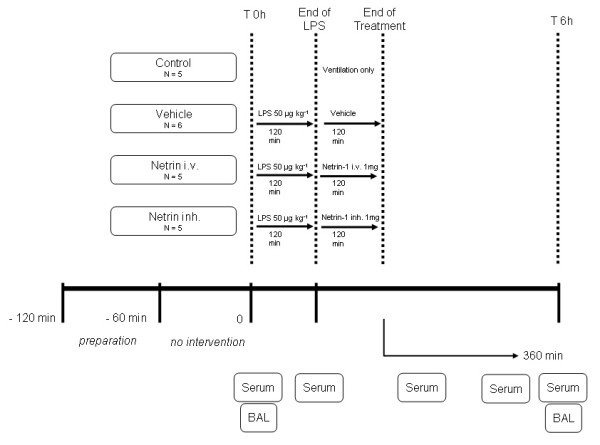
**Experimental protocol**. Control animals received mechanical ventilation only. Acute lung injury (ALI) was induced through lipoploysaccharide (LPS) infusion (50 μg/kg over 2 h), and subsequently animals were treated with either vehicle (50 ml of NaCl 0.9% + 1 mg of bovine serum albumin), netrin-1 i.v. (1 mg i.v. in 50 ml of NaCl 0.9%) or netrin-1 inh. (1 mg in 50 ml of NaCl 0.9%) for 2 hours. Following this, the observation period was 6 hours until the end of the experimental protocol. Hemodynamic measurements, serum levels of cytokines and bronchoalveolar lavage (BAL) were determined as indicated.

*Escherichia coli *055:B5 lipopolysaccharide (Sigma Chemicals, Munich, Germany) was infused at a concentration of 50 μg kg^-1 ^for 2 hours. Animals were divided into four groups: (1) no LPS infusion, (2) LPS infusion + vehicle treatment, (3) LPS infusion + netrin-1 i.v. (1 mg over 2 hours) and (4) LPS infusion + netrin-1 inh. (1 mg over 2 hours). For further details about the experimental protocol, see Figure 1.

### BAL myeloperoxidase activity and protein content

BAL supernatant was used for protein measurements performed according to standard methods. Pulmonary myeloperoxidase activity was quantified by enzymatic assay for the azurophilic neutrophil granule protein myeloperoxidase (MPO) as described previously [26].

### Histopathological evaluation of ALI

After animals were killed, lung tissue was excised from the right lower lobe of the lung and fixed in 10% formaldehyde solution. Tissues were then embedded in paraffin and stained with hematoxylin and eosin. Three random tissue sections from four different lungs in each group were examined.

### Application of Netrin-1

Inhaled netrin-1 (1 mg) was dissolved in 20 ml of NaCl 0.9% and applied through Venta-neb-ir (NEBU TEC, Elsenfeld, Germany) over a period of 2 hours. Intravenous netrin-1 (1 mg) was dissolved and applied with a standard infusion pump through a central venous catheter. Vehicle control was performed using porcine serum albumin (1 mg; Sigma Chemicals, Munich, Germany) dissolved in 20 ml of NaCl 0.9% and applied nebulized and intravenously simultaneously. Calculation of netrin-1 dose resulted from experiments previously performed in mice [20] and was calculated according to porcine weight. Given the fact that netrin i.v. and netrin inh. were equally efficient in our murine model, we also used an equal dose of netrin-1 i.v. and netrin-1 inh. in this porcine model.

### Measurement of BAL cytokine concentration

Cytokine concentration was measured in supernatant of BAL and in serum of animals with standard enzyme-linked immunosorbent assay kits (R&D Systems, Minneapolis, MN, USA).

### Computer tomography

At the end of the study protocol, animals were killed and transported immediately to a standard spiral computer tomographer (Siemens, Munich, Germany). Animals were then scanned using standard protocol for evaluation of radiographic signs of lung injury.

### Statistical analysis

Data are given as means ± standard error of the mean. Statistical analysis was carried out using the JMP software package (SAS, Cary, NC, USA). Values were analyzed by Mann-Whitney *U *test for independent groups, since normal distribution of samples was not assumed (SigmaStat 3.10; Systat Software, San Jose, CA, USA). Statistical significance was assumed at *P *< 0.05.

## Results

### Hemodynamic measurements do not differ between intervention groups

Following the experimental protocol (Figure 1), we performed systemic hemodynamics, pulmonary hemodynamics and other variables to evaluate whether we could determine a significant difference between groups. We found a significant increase of systemic vascular resistance, mean arterial pressure, pulmonary vascular resistance, mean pulmonary pressure, pulmonary capillary wedge pressure and cardiac index in animals following LPS infusion, which was performed as described previously [27]. However, we did not find a significant difference between the vehicle, the netrin-1 i.v. or netrin-1 inh. groups (Table 1).

### Evaluation of pulmonary injury

To identify the role of LPS infusion on pulmonary oxygenation and ventilation, we evaluated variables determining changes in respiration, oxygenation and ventilation. We found decreased static compliance and an increase in inspiratory pressure after LPS exposure. Furthermore, we found that animals treated with netrin-1 had a tendency for an improved oxygenation index compared to animals in the vehicle group (vehicle, 158 ± 30; netrin-1 i.v., 204 ± 31; netrin-1 inh., 217 ± 20; *P *= 0.09) (Table 2).

**Table 2:** Variables determining respiration, oxygenation and ventilation in vehicle controls and netrin-1-treated animals

Parameter	Group		Time	
		
		0 h	4 h	6 h
PaO_2_/FiO_2 _(mmHg)	Control	483 ± 31	513 ± 48	493 ± 77
	LPS + Vehicle	453 ± 10	255 ± 38*	158 ± 30*
	LPS + Netrin i.v.	491 ± 31	254 ± 18*	204 ± 31*
	LPS + Netrin inh	487 ± 6	272 ± 11*	217 ± 20*

PaCO_2 _(kPa)	Control	5.2 ± 0.2	5.1 ± 0.2	4.8 ± 0.1
	LPS + Vehicle	5.0 ± 0.1	5.1 ± 0.2	5.4 ± 0.3
	LPS + Netrin i.v.	5.3 ± 0.4	5.1 ± 0.2	5.2 ± 0.7
	LPS + Netrin inh.	5.0 ± 0.2	4.6 ± 0.1	5.2 ± 0.3

DO_2 _(ml min^-1 ^kg^-1^)	Control	1207 ± 60	1139 ± 49	940 ± 88
	LPS + Vehicle	1132 ± 61	1132 ± 97	1009 ± 28
	LPS + Netrin i.v.	1207 ± 83	1139 ± 97	960 ± 69
	LPS + Netrin inh	1102 ± 38	1147 ± 120	997 ± 84

P_peak _(mbar)	Control	14.4 ± 0.4	14.5 ± 0.4	14.7 ± 0.8
	LPS + Vehicle	14.4 ± 0.5	20.8 ± 2.1*	22.7 ± 3.0*
	LPS + Netrin i.v.	14.4 ± 1.0	18.9 ± 1.4*	19.8 ± 1.8*
	LPS + Netrin inh	14.2 ± 0.4	19.2 ± 0.2*	20.7 ± 0.7*

P_mean _(mbar)	Control	8.1 ± 0.5	8.2 ± 0.5	8.4 ± 0.7
	LPS + Vehicle	7.7 ± 0.2	10.3 ± 1.2	11.5 ± 1.8
	LPS + Netrin i.v.	7.4 ± 0.2	9.3 ± 0.3	9.3 ± 0.3
	LPS + Netrin inh.	7.6 ± 0.2	9.3 ± 0.1	10.1 ± 0.3

Compl.st. (ml cm H_2_O^-1^)	Control	28.3 ± 1.2	29.4 ± 1.0	29.5 ± 1.2
	LPS + Vehicle	28.6 ± 0.7	19.3 ± 1.6*	18.2 ± 1.7*
	LPS + Netrin i.v.	28.4 ± 2.0	19.9 ± 1.4*	19.3 ± 1.7*
	LPS + Netrin inh	27.7 ± 1.4	19.1 ± 0.7*	17.7 ± 0.7*

Measurements determining the oxygenation and ventilation in control animals, in vehicle animals and animals subjected to netrin-1 4 hours and 6 hours after the end of treatment (data are means ± SEM and indexed in body weight; *P *< 0.05; *versus control group). Horovitz Index (OI), inspiratory oxygen fraction (FiO_2_), carbon dioxide partial pressure (PaCO_2_), systemic oxygen delivery (DO_2_), peak airway pressure (P_insp_), mean airway pressure (P_mean_), static compliance (Compl.st.), respiratory frequency (RF).

### Animals treated with netrin-1 demonstrate reduced radiological signs of ALI

**Figure 2:**
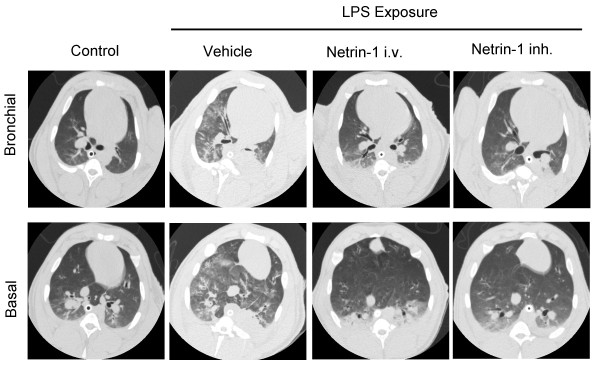
**Netrin-1 dampens radiographic signs of ALI**. Chest computed tomography was performed in animals after mechanical ventilation (control) 6 hours following treatment with vehicle, netrin-1 i.v. or netrin-1 inh. Pictures demonstrate one representative animal per group (*n *= 4 per group).

Initially, we evaluated whether the animals treated with netrin-1 would demonstrate reduced radiological signs of pulmonary injury after 6 hours compared to vehicle controls. For this purpose, we performed standard spiral chest computed tomography (CT) and evaluated pulmonary injury at the level of the bronchi just below the tracheal bifurcation and at the basal level just above the diaphragm. We found reduced signs of pulmonary edema, patchy infiltrates and inflammatory changes in the animals treated with netrin-1 i.v. or netrin-1 inh. as compared to vehicle controls (Figure 2). Control animals receiving mechanical ventilation only did not demonstrate significant pulmonary changes.

### Intravenous netrin-1 dampens systemic inflammatory cytokines

**Figure 3:**
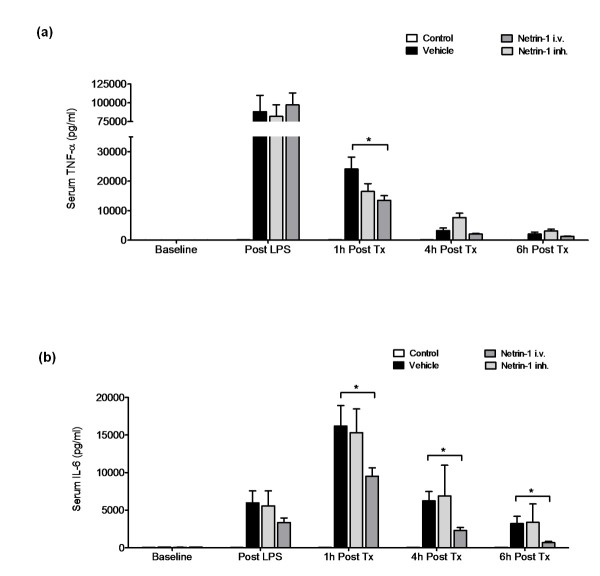
**Netrin-1 dampens systemic inflammation**. **(a) **Serum tumor necrosis factor (TNF)-α levels and **(b) **serum interleukin (IL)-6 levels in controls prior to treatment and 6 hours following treatment with vehicle, netrin-1 i.v. or netrin-1 inh. (data are means ± SEM; *P < 0.05 as indicated, *n *= 5-6 per group).

To identify whether netrin-1 administration would result in a significant reduction of systemic inflammation, we evaluated levels of serum TNF-α and interleukin-6. For this purpose, we obtained blood samples at the termination of LPS infusion, 1 hour after the initiation of netrin-1 exposure and 4 hours and 6 hours after netrin-1 administration. We found a significant reduction of serum TNF-α 1 hour after administration of netrin-1 i.v. When determining the levels of interleukin-6, we found a consistent reduction of interleukin-6 at 1, 4 and 6 hours in the netrin-1 i.v. group compared to the vehicle group (Figure 3). No significant effect was observed in the netrin-1 inh. group.

### Netrin-1 reduces alveolar cytokine levels

**Figure 4:**
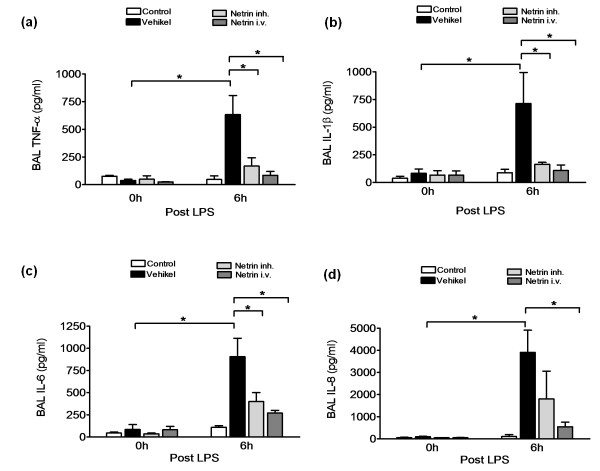
**Netrin-1 dampens pulmonary cytokine release**. **(a) **TNF-α levels in bronchoalveolar lavage (BAL) of control animals, animals treated with vehicle netrin-1 i.v. or netrin-1 inh. **(b) **Concentration of interleukin-1β in the BAL. **(c) **Concentration of interleukin-6 in the BAL. **(d) **Concentration of interleukin-8 in the BAL prior to treatment and 6 hours following treatment with vehicle, netrin-1 i.v. or netrin-1 inh. (data are means ± SEM; *P < 0.05 as indicated, *n *= 5-6 per group).

To identify whether the anti-inflammatory effect of netrin-1 would influence the inflammatory process in the alveolar compartment of the lung, we performed bronchoalveolar lavage and determined the levels of inflammatory cytokines within this lavage. We found significant reduced levels of TNF-α (vehicle, 633 ± 172 pg ml^-1^; netrin-1 i.v., 84 ± 5 pg ml^-1^; netrin-1 inh., 168 ± 74 pg ml^-1^; both *P *< 0.05), interleukin-1β (vehicle, 713 ± 280 pg ml^-1^; netrin-1 i.v., 108 ± 5 pg ml^-1^; netrin-1 inh., 164 ± 18 pg ml^-1^; both *P *< 0.05) and interleukin-6 (vehicle, 906 ± 207 pg ml^-1^; netrin-1 i.v., 269 ± 31 pg ml^-1^; netrin-1 inh., 399 ± 101 pg ml^-1^; both *P *< 0.05) in the BAL of the animals treated with netrin-1 compared to the vehicle group (Figures 4a-c). When determining the concentration of interleukin-8 within the BAL, we found a significant reduction in the netrin-1 i.v. group and a trend toward a reduction in the netrin-1 inh. group (Figure 4d).

### Netrin-1 reduces protein content, MPO activity and histopathological signs of ALI

**Figure 5:**
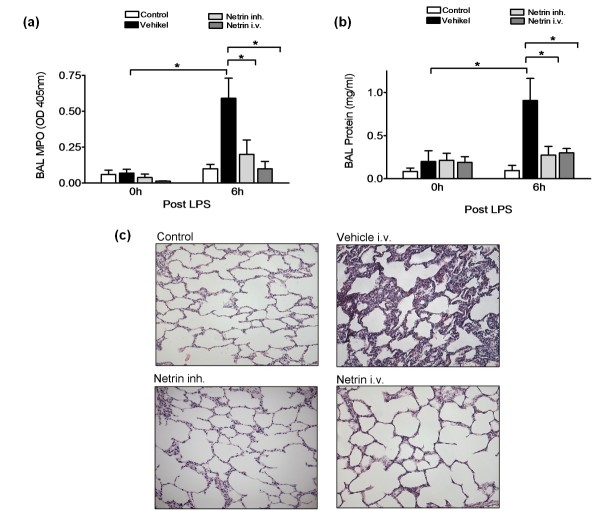
**Netrin-1 dampens myeloperoxidase activity and histopathological signs of pulmonary inflammation**. **(a) **Myeloperoxidase activity in the BAL of control animals, animals treated with vehicle after LPS infusion and animals treated with either netrin-1 i.v. or netrin-1 inh. **(b) **Protein content in the BAL of experimental animals. **(c) **Histology sections of controls and 6 hours following treatment with vehicle, netrin-1 i.v. or netrin-1 inh. (data are means ± SEM; *P < 0.05 as indicated, *n *= 5-6 per group, histology sections are from one representative animal per group).

Next, we proceeded to investigate whether netrin-1 influences the content of protein within the alveolar space and the leukocyte activity measured through the activity of MPO. Netrin-1 i.v. and netrin-1 inh. reduced the protein content and MPO activity within the alveolar space (Figures 5a and 5b). This was reflected in the histopathological sections, demonstrating reduced histological signs of inflammation in the netrin-1 treatment groups (Figure 5c).

## Discussion

We report here that the neuronal guidance protein netrin-1 significantly dampens the degree of intra-alveolar inflammation in a porcine model of lung injury. Netrin-1 administration resulted in a reduction of intra-alveolar MPO activity, reduced alveolar protein content and improved histological appearance of pulmonary tissue. Furthermore, we found that netrin-1 administration reduced the concentration of inflammatory cytokines within the alveolar space. As such, netrin-1 dampened a crucial component of lung injury, since pulmonary inflammation is the major contributor to the pathological derangement of ALI.

The initial stages of ALI are marked by the infiltration of neutrophils from the vascular into the alveolar space, which becomes the site of a profound inflammation [28]. Proinflammatory cytokines such as TNF-α or interleukin-8 foster this intra-alveolar inflammation, which might subsequently become self-propagating and persist long after the initial pulmonary insult is resolved. The clinical prognosis of ALI correlates with the extent of this intra-alveolar inflammation [14,15]. This is underscored by the fact that the concentration of inflammatory cytokines is reduced in patients surviving ARDS compared to nonsurvivors [29]. Current therapy strategies are aimed to achieve control of this pulmonary inflammation though antimicrobial therapy or the modification of mechanical ventilation [30,31]. In this study, we attempted a novel approach for anti-inflammatory control and used the neuronal guidance protein netrin-1 in a large animal model. As such, we moved from a murine wild-type and knockout model, which focused on the mechanisms underlying the anti-inflammatory properties of netrin-1, toward the porcine LPS-induced ALI model that more closely resembles the human pathobiology. We were able to confirm in this model the potential of netrin-1 to reduce intra-alveolar inflammation, which was associated with a tendency of netrin-1 to improve oxygenation. Furthermore, alveolar cytokine levels were significantly reduced through netrin-1 treatment. Therefore, our findings demonstrate that a significant reduction of the inflammatory changes of ALI is possible though the neuronal guidance protein netrin-1 and confirm our previous findings in mice [20].

Only recently neuronal guidance proteins have been discovered to hold anti-inflammatory potential [18,19,26]. The guidance protein netrin-1 serves as a stop signal during axonal growth in the CNS [32,33]. Subsequent work has demonstrated the expression of netrin-1 outside the CNS and the importance of netrin-1 for neutrophil migration [19]. Netrin-1 is expressed in large quantities in the endothelium, where it induces angiogenesis and controls the trafficking of leukocytes from the vascular space [19,34]. Furthermore, netrin-1 possesses anti-inflammatory and tissue-protective potential during renal and myocardial ischemia-reperfusion (IR) injury [35,36]. Netrin-1 is also expressed in significant amounts by epithelial cells on mucosal surfaces such as the intestine and the lung [26]. We have demonstrated previously that pulmonary netrin-1 expression is reduced during inflammatory or mechanical lung injury and that substitution with exogenous netrin-1 results in reduced pulmonary inflammation through the A2BAR. In this study, we attempted to further these findings one step closer to clinical application and administered netrin-1 i.v. or netrin-1 inh. in a porcine model. We found in this study that the netrin-1 i.v. application possesses greater anti-inflammatory potential compared to netrin-1 inh. This was somehow surprising to us, given that we previously found an equal potential of intravenous and inhaled netrin-1 administration to reduce pulmonary inflammation in our murine study. This finding is likely explained by the fact that netrin-1 i.v. is more efficient owing to the safe and controlled administration of netrin-1 via intravenous infusion compared to the netrin-1 inh. application that carries the limitations of nebulized drug administration during mechanical ventilation. No drugs are currently available to induce netrin-1 selectively, but this could be the focus of future research and would avoid the administration of the expensive recombinant protein.

Several other limitations of the presented study have to be outlined. First, we did not confirm the reduction of netrin-1 within pulmonary tissue, since the netrin-1 sequence in the porcine species used is not available. Given the fact that both Ly *et al*. [19] and Mirakaj *et al*. [20] demonstrated a reduction of netrin-1 within pulmonary tissue during lung injury, it has to be considered likely that a repression of netrin-1 might also be present in this model of ALI. Second, we did not demonstrate a survival benefit in the study group receiving netrin-1 treatment compared to the vehicle group. Several studies have used porcine models of ALI to report important novel findings, yet have not reported the associated mortality benefit of their intervention [37,38]. During a potential long-term ventilation model, repeated administration of netrin-1 would be necessary to prove a benefit of netrin-1 for survival. Given the extremely high cost of such an intervention at the moment, future well-funded studies will be necessary to answer this question. Finally, the small number of animals used has to be seen as a limitation of this study. The primary focus of this study, however, was to highlight a novel approach for the control of intra-alveolar inflammation through a neuronal guidance protein in a large animal model, and this is described in this study for the first time. The tendency for improved oxygenation in both netrin-1 groups is most likely a reflection of this reduced intra-alveolar inflammation, as we also found a significant reduction of intra-alveolar cytokine levels and improved histology in these groups. Therefore, on the basis of our data, although limited through the number of animals studied, we propose to pursue this novel substance as a potential future therapeutic agent for conditions associated with lung injury.

Taken together, the present study implicates a novel approach for the control of pulmonary inflammation during the initial stages of ALI. Additional studies are needed to further explore the therapeutic potential of netrin-1 during acute inflammatory changes in the lung, thereby leading the way to a possible translation from animal studies toward novel therapies for ALI in humans.

## Conclusions

In this study, we addressed our recent finding of the anti-inflammatory role of netrin-1 and used inhaled or intravenous netrin-1 to reduce intra-alveolar inflammation during ALI in a porcine model. ALI is marked by a self-propagating inflammation within the alveolar space that results in the loss of pulmonary function. Therapeutic strategies are aimed at reducing this inflammatory process. We present here evidence that the application of netrin-1 results in reduced leukocyte activity within the alveolar space, dampens the release of cytokines and improves the histolopathological changes of pulmonary tissue in animals exposed to netrin-1. This study therefore brings one step forward the only recently discovered anti-inflammatory function of netrin-1 to a potential therapeutic translation and application for the treatment of ALI.

## Key messages

• Inhaled netrin-1 dampens the extent of intra-alveolar inflammation during ALI.

• Intravenous application of netrin-1 additionally possesses the potential to reduce systemic inflammation measured as serum cytokine concentration during ALI.

• The histological and radiological pathology of ALI are attenuated through netrin-1.

• To the best of our knowledge, this study is the first to report the potential of recombinant netrin-1 in a large animal model and enables a possible translation of the research findings from mice to humans.

## Abbreviations

ALI: acute lung injury; ARDS: acute respiratory distress syndrome; BAL: bronchoalveolar lavage; CNS: central nervous system; CT: computed tomography; CVP: central venous pressure; DO_2l_: systemic oxygen delivery; EVWL: extravascular lung water; H&E: hematoxylin and eosin; HR: heart rate; IL: interleukin; inh., inhaled; ITBV: intrathoracic blood volume; i.v.: intravenous; MAP: mean arterial pressure; LPS: lipopolysaccharide; MPO: myeloperoxidase; PCWP: pulmonary capillary wedge pressure; PAP: pulmonary artery pressure; PVR: pulmonary vascular resistance; SVR: systemic vascular resistance; TNF: tumor necrosis factor.

## Competing interests

The authors declare that they have no competing interests.

## Authors' contributions

The hypothesis was developed by PR and VM; animal experiments were performed by CM, PW, and KW; and *in vitro *analysis was performed by KK and VM. The manuscript was written and edited by DAV, TI, GNS and PR.
